# Rapid onset and unpredicted findings of a giant nodular melanoma arising on a congenital nevus: a case report

**DOI:** 10.1093/omcr/omac128

**Published:** 2022-11-24

**Authors:** Alin Mallouhy, Edwar Kounsselie, Amira Bitar, Ebrahim Makhoul, Moatasem Hussein Al-janabi, Zuheir Alshehabi, Michael Georgeos

**Affiliations:** Department of Radiology, Tishreen University Hospital, Latakia, Syria; Cancer Research Center, Tishreen University Hospital, Latakia, Syria; Cancer Research Center, Tishreen University Hospital, Latakia, Syria; Cancer Research Center, Tishreen University Hospital, Latakia, Syria; Department of Pathology, Cancer Research Center, Tishreen University Hospital, Latakia, Syria; Department of Pathology, Cancer Research Center, Tishreen University Hospital, Latakia, Syria; Department of Oncology, Tishreen University Hospital, Latakia, Syria

## Abstract

Melanoma is a malignancy that develops from pigment-producing cells known as melanocytes. Although it is considered one of the most fatal tumors in the world, its early diagnosis is associated with a better prognosis and overall survival. A 49-year-old man was admitted to the dermatology department with a 15 cm lesion on the left arm. It was growing on a congenital nevus to reach an enormous size. Surgical excision was not possible and the treatment was controversial as the patient showed unpredicted resistance to immunotherapy. Further evaluations revealed false-negative BRAF mutation, which completely changed the course of treatment. Cutaneous melanoma is a rare malignancy, accounting only for 1% of skin cancer cases, and having it arising on a pre-existing congenital nevus is even much rarer. Although there is no decisive definition of giant melanoma, some authors define it as a lesion of more than 10 cm in diameter. Through the literature, only a few cases of giant melanoma on the arm have been reported. Through our paper, we are revealing the importance of early diagnosis and treatment of melanoma and confirming the significant role of regular follow-up for patients with a congenital melanocytic nevus. Moreover, we are showing the importance of having alternative methods for detecting BRAF mutations to avoid false-negative results and have better outcomes.

## INTRODUCTION

Malignant melanoma represents only about 1% of all skin cancers and <1% grows on a congenital nevus [1]. However, melanoma is an aggressive tumor and its incidence is increasing. It usually tends to grow rapidly but rarely measures more than 10 cm [2]. In our case, we reported a giant pigmented nodular melanoma that was neglected and disregarded, and it grew on a congenital nevus.

## CASE REPORT

**Figure 1:**
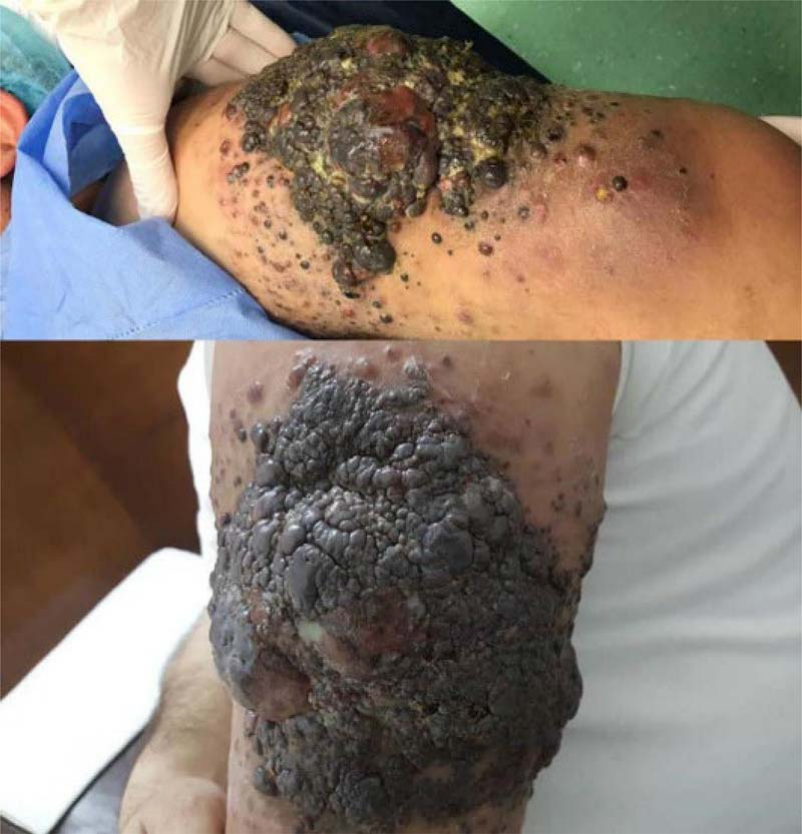
Clinical image showing a giant cutaneous lesion, vegetative and erupted with irregular borders lesion.

A 49-year-old male was presented to our clinic with a wide ulcerated skin lesion on the upper area of the left arm. It started 6 months earlier on a congenital nevus, which remained unchanged since birth. There were no skin tumors in his family history. Laboratory tests were within the normal range. Physical examination showed a reddish-brown to black-colored, vegetative and erupted with irregular borders lesion. It measured 15 cm × 12 cm × 2 cm in the left deltoid area (Fig. 1).

There were many differential diagnoses. Nodular melanoma appears as an amelanotic symmetrical and well-circumscribed papule or nodule with a uniform color, although it can be melanotic and ulcerated occasionally [3]. Lentigo maligna melanoma manifests as an irregular pigmented large patch or macule on sun damaged areas of the skin or as hypomelanotic/amelanotic papule [4]. It arises from spindle-shaped junctional melanocytes [5]. Superficial spreading melanoma presents as a scalloped, asymmetric and irregular papule or macule arising on previous nevi or developed *de novo* [6].

**Figure 2:**
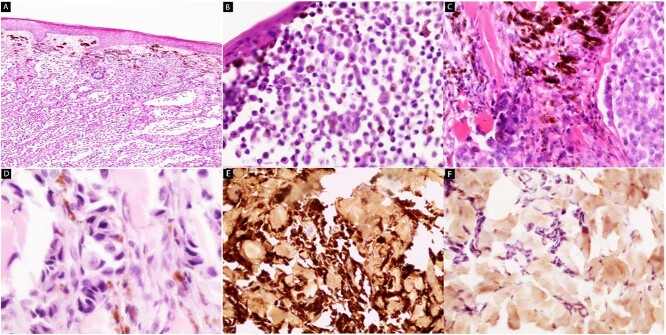
Hematoxylin and eosin stain (**A**–**D**) and IHC (**E** and **F**): Microscopic images of cutaneous biopsy. (A) Nodular dermal proliferation of melanocytes in the dermis (×40). (B) Infiltrated atypical polymorphic melanocytes with lymphoid inflammatory cells (×100). (C) Coarse brown cytoplasmic pigment is seen (×100). (D) Malignant cells showed large hyperchromatic atypical nuclei with basophilic cytoplasm (×400). (E) The tumor cells are positive for melanoma cocktail (×100). (F) Pan-cytokeratin (CK) is negative (×100).

A cutaneous biopsy was performed and sent to the pathology department. Microscopic examination of the biopsy revealed proliferation of variably size nodules of malignant cells in the dermis (Fig. 2A). Infiltrated atypical polymorphic melanocytes with mild lymphoid inflammatory cells (Fig. 2B) and coarse brown pigments were observed (Fig. 2C). The malignant cells had large hyperchromatic nuclei with basophilic cytoplasm (Fig. 2D). To confirm the diagnosis, immunohistochemical stains were applied. The tumor cells were positive for melanoma cocktail (Fig. 2E) and were negative for pan-cytokeratin (Fig. 2F). We did not perform dermoscopy because of its size and necrotic status, which pushed us to do the biopsy as early as possible.

**Figure 3:**
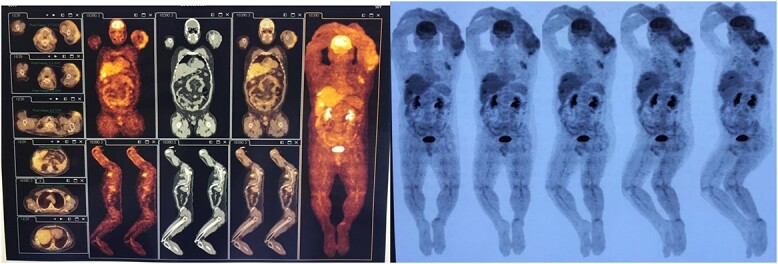
Positron emission tomography-computed tomography scan revealed hypermetabolic lesions. It shows local and distant metastases to the lymph nodes, liver and bone.

By performing the radiological investigation, positron emission tomography-computed tomography scan revealed hypermetabolic lesions with local and distant metastases to the lymph nodes, liver and bone (Fig. 3), while magnetic resonance imaging (MRI) of the brain was within normal limits.

**Figure 4:**
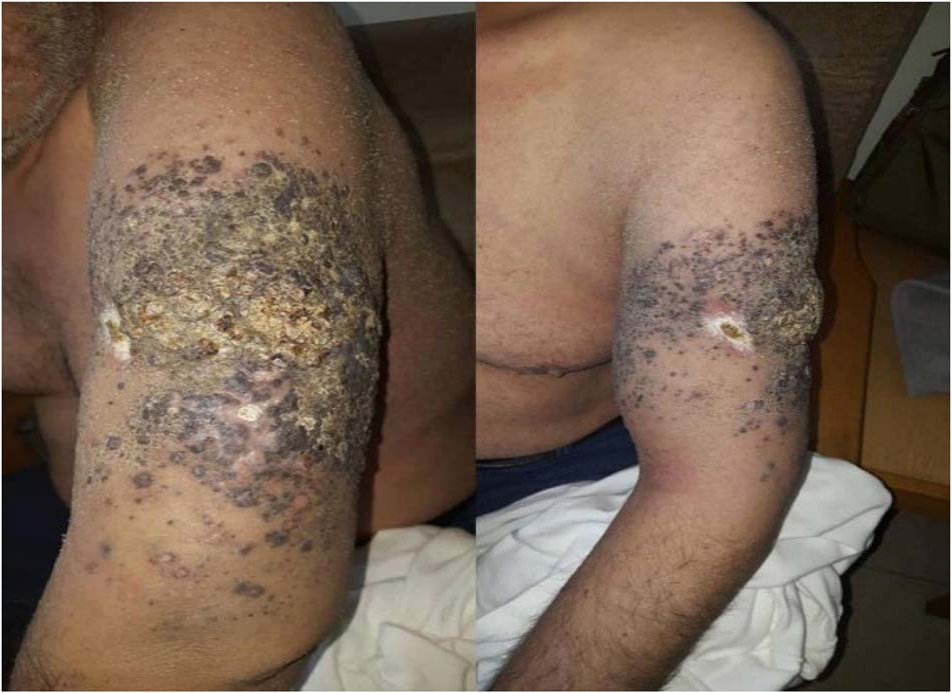
Clinical image shows very good response and the lesion has regressed properly after 1 month of targeted therapy.

BRAF mutation analysis was performed and the result was negative for BRAF V600E. Therefore, targeted therapy with anti-BRAF drugs was excluded and the patient underwent chemotherapy with dacarbazine 250 mg/m^2^ D1 → D5. A good response started after two courses but the patient had rapid progression after initiating the third course, which encouraged us to change the protocol to paclitaxel 100 mg/m^2^ D1, D8, D15 and carboplatin 150 mg/m^2^ D1, D8, D15, but there was no response. At that stage, the patient suffered from disabled left arm due to severe pain and edema. Immunotherapy with nivolumab 280 mg/m^2^ was initiated, but unfortunately, there was no response observed after three courses. Surgical consultation claimed that surgical intervention is inapplicable due to locally advanced disease. Another wedge biopsy was taken for further histopathology evaluation. The result was identical to the first biopsy. The tumor was invading to anatomic level (at least V-Clark) and a measured depth (at least 3.5 cm-Breslow). The second BRAF mutation analysis, in contrast to the first one, came positive for BRAF V600E mutation. Targeted therapy with vemurafenib 960 mg was initiated. A significant response was noted within 2 weeks, including the appearance (Fig. 4), edema, pain and distant metastases. Three months later, a brain MRI showed multiple lesions in both brain lobes and the brainstem with altered mental status and confusion. Unfortunately, the patient deteriorated rapidly and died due to complications of his brain metastases.

## DISCUSSION

Cutaneous melanoma is a rare malignancy, accounting only for 1% of skin cancer cases, and having it arising on a pre-existing congenital nevus is even much rarer [1]. Although there is no decisive definition of giant melanoma, some authors define it as a lesion of more than 10 cm in diameter [2]. Through the literature, only a few cases of giant melanoma on the arm have been reported. [7]. 50–70% of melanomas have positive BRAF mutation [8]. Better survival and a quicker response to treatment in patients with positive BRAF mutation had been reported after the development of the BRAF inhibitors and MEK inhibitors [9]. The BRAF gene organizes and adjusts the division and senescence of the cells to prevent the proliferation of cancer precursor cells, by encoding a protein kinase of the mitogen-activated protein kinase pathway. The deregulation of the MAPK signal transduction pathway occurs when the BRAF mutation gets activated [10]. V600E type is the most observed BRAF mutation in clinical practice [9]. In our case, the lesion has been neglected by the patient, evolving rapidly to reach an enormous size. Such growing on a congenital nevus may have been a contributing factor in the ignorance of this lesion. A false-negative BRAF mutation result, using polymerase chain reaction, led us to exclude targeted therapy. Unresponsiveness to immunotherapy was unjustified, which guide us to order a second BRAF mutation analysis, and in contrast to the first one, it came positive for BRAF mutation. False-negative BRAF mutation results can be a threatening problem, eventuating in delayed treatment and poor outcomes. However, immunohistochemistry (IHC) using VE1, unlike DNA-based techniques, recognizes the therapeutic target protein and estimates its expression in malignant cells [9]. Moreover, IHC is less expensive, can be used on fewer tumor cells, is simpler and has faster results compared to DNA-based techniques [8]. However, IHC using VE1 can only detect the V600E mutation. Therefore, combining both, DNA-based techniques and IHC using VE1 can lead to more accurate results in BRAF mutation analysis [9].

## CONCLUSION

Cutaneous melanoma is a very aggressive type of skin cancer that can arise from a pre-existing congenital nevus. Our case reveals the significant role of regular follow-up on any pigmented skin lesions, especially melanocytic nevus. Furthermore, it indicates the importance of having alternative methods for detecting BRAF mutations to avoid false-negative results and therefore have better outcomes.

## CONFLICT OF INTEREST STATEMENT

The authors have no conflicts of interest to declare.

## FUNDING

None.

## REGISTRATION OF RESEARCH STUDIES

Not applicable.

## CONSENT

Written informed consent was obtained from the patient for the publication of this case report. A copy is available for review by the Editor-in-Chief of this journal.

## ETHICAL APPROVAL

No approval was required for this submission.
